# Association between cardiometabolic index and congestive heart failure among US adults: a cross-sectional study

**DOI:** 10.3389/fcvm.2024.1433950

**Published:** 2024-09-10

**Authors:** Xi Luo, Bin Cai

**Affiliations:** ^1^ Department of Clinical Nutrition, Tongde Hospital of Zhejiang Province, Hangzhou, China; ^2^ Department of Clinical Nutrition, Sir Run Run Shaw Hospital, Zhejiang University School of Medicine, Hangzhou, China; ^3^ Department of Clinical Nutrition, Shaoxing People’s Hospital, Shaoxing, China

**Keywords:** congestive heart failure, age, cardiometabolic index, visceral fat, NHANES

## Abstract

**Background:**

The risk of congestive heart failure (CHF) is significantly affected by obesity. However, data on the association between visceral obesity and the risk of CHF remain limited. We explored the relationship between CHF and cardiometabolic index (CMI).

**Methods:**

Drawing from the National Health and Nutrition Examination Survey (NHANES) for 2011–2018, we enrolled 9,008 participants in a cross-sectional study. We calculated the CMI as triglyceride (TG)/high density lipid-cholesterol (HDL-C) × weight-to-height ratio (WHtR), and CMI-age as CMI × age. Then, we analyzed CMI and CMI-age as categorical and continuous variables to assess its correlation with CHF. To assess the relationships of CMI and CMI-age with CHF, we used multiple logistic regression models and performed subgroup analysis. To examine the predictive ability of CMI and CMI-age on patients with CHF, we used receiver operating characteristic (ROC) curves.

**Results:**

The overall prevalence of CHF was 3.31%. The results revealed significant differences in demographic data, comorbidities, lifestyle variables, standing height, BMI, WC, WHtR, TG, and HDL-C among the four groups classified by CMI quartile and CMI-age quartile. When indicators were analyzed as continuous variables, CMI and CMI-age showed positive correlations with CHF in both the crude and adjusted models (all *P* < 0.05). When indicators were analyzed as categorical variables, it was found that in all four models, the ORs of group Q4 was significantly different compared to Q1 (all *P* < 0.05), suggesting the risk of CHF is significantly increased with higher CMI, and CMI-age. The associations of CMI and CMI-age with CHF were similar in all stratified populations (*P* for interaction > 0.05). The areas under the ROC curve (AUCs) of CMI and CMI-age in predicting CHF were 0.610 (95% CI, 0.578–0.642) and 0.697 (95% CI, 0.668–0.725) separately, suggesting that CMI-age was significantly better than the CMI in predicting CHF (*P *< 0.001).

**Conclusions:**

Both CMI and CMI-age were independently correlated with the risk for CHF. These results suggested that the CMI-age, which provides new insights into the prevention and management of CHF. CMI-age could serve as effective tools to identify CHF during primary care examinations and in medically resource-limited areas.

## Introduction

Functional and structural impairments of ventricular filling or blood ejection characterize the symptoms of congestive heart failure (CHF) (1). This life-threatening syndrome is responsible for significant morbidity and mortality as well as for limited quality of life and functional ability. High costs are also attributed to this complex syndrome (2, 3). Because of aging populations, the number of CHF cases is increasing, and to date, more than 64 million people suffer from CHF globally. Comorbidities and associated risk factors as well as longer survival times after myocardial infarction are also contributing to this rise in cases (2–4). Recent literature has also indicated that the HF burden in 50 years or younger is increasing (4). Hence, the early detection and aggressive modification of risk factors for CHF are of immense significance to prevent progression.

Obesity not only directly harms the myocardium but also indirectly increases the risk of CHF by promoting the development of many metabolic risk factors (5–7). Studies have revealed that the marked rise in prevalence of obesity, which is most prominent among those from impoverished socioeconomic backgrounds, has been among the strong risk factors for the development of CHF (7). Although body mass index (BMI) is very common to describe the obesity-related cardiovascular risk, it may not fully reflect the amount and distribution of body fat and fail to differentiate the increased body fat content, preserved or increased lean mass, and the body hydration status (6–8). Compared to obesity defined by BMI, the accumulation of visceral adipose is widely recognized as a more accurate predictor of morbidity and mortality in CHF (6, 7). To assess visceral obesity, a new simple and noninvasive index that combines weight-to-height ratio (WHtR) and biochemical lipid parameters has been suggested, called the cardiometabolic index (CMI) (9). Studies have shown a relationship between the CMI and diabetes mellitus (DM), cardiovascular disease (CVDs), and metabolic syndrome (MetS), suggesting that the CMI is useful for screening for these conditions (9–15). Nevertheless, there has been no investigation into the possible correlation between CMI and CHF, nor has it been tested as a screening tool for the condition. Furthermore, according to data from the European Society of Cardiology, 10% CHF patients are over 70 years old, and thus a combination of CMI with age may potentially identify CHF more strongly than CMI (16). Therefore, the aim of this study was to evaluate the association of CMI and CMI-age with CHF, and to compare the two indicators for early identification of CHF using the data of the National Health and Nutrition Examination Survey (NHANES) from 2011 to 2018.

## Materials and methods

### Study population

**Figure 1:**
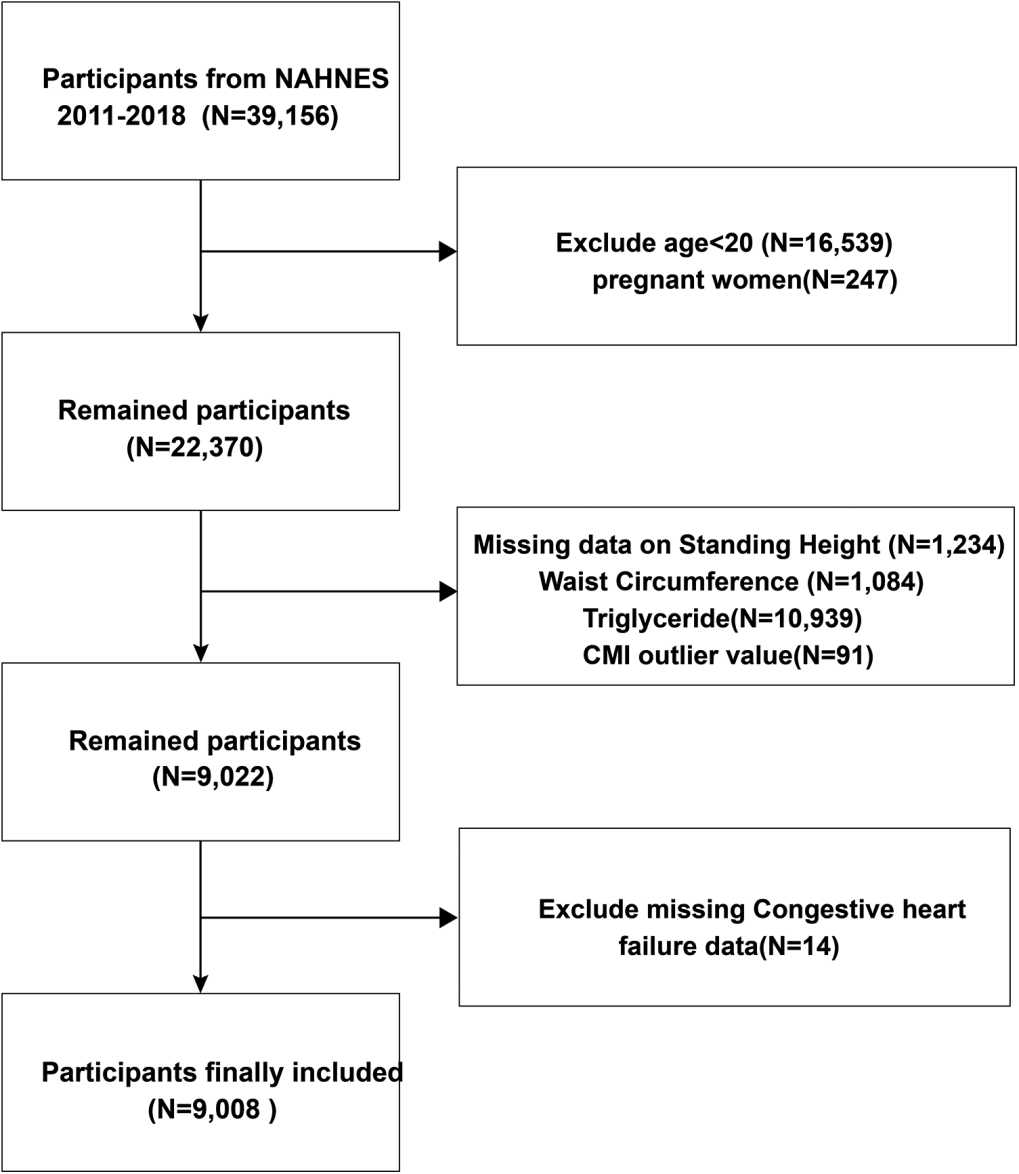
Flow chart of study participants. CMI, cardiometabolic index.

This survey collected data including demographics, physical examinations, questionnaire, and health-related data from the NHANES database for 2011–2018. All survey methods and data are available at https://www.cdc.gov/nchs/nhanes/about_nhanes.htm. The National Center for Health Statistics Research Ethics Review Board approved the research protocols. Our exclusion criteria included age < 20 years, missing CHF status, missing CMI-related parameters, and pregnancy (Figure 1).

### Definitions

The verification of CHF is based on the questionnaire from MCQ, similar to published NHANES-based articles (14). Participants were asked the following question: “has a doctor ever told you that you have CHF?” Those who responded “yes” were classified as having CHF. The criteria for hypertension included self-reported physician-diagnosed hypertension, antihypertensive drug use, or systolic and diastolic blood pressure (BP) of ≥140 or ≥90 mmHg (6). The criteria for self-reported or physician-diagnosed diabetes mellitus included (1) 126 mg/dl of fasting glucose or 200 mg/dl of plasma glucose within 2 h of taking an oral glucose tolerance test, and (2) the use of insulin or oral hypoglycemic medications (6). We used the following formulas: BMI (kg/m^2^) = weight/height squared. WHtR = waist circumference (WC, cm)/height (cm). CMI = Triglyceride (TG, mmol/L)/high-density lipoprotein-cholesterol (HDL-C, mmol/L) × WHtR (9). CMI-age = CMI × age.

### Covariates

We obtained the particular methodologies and caliber of determination for every covariate control approach from NHANES (https://www.cdc.gov/nchs/nhanes/about_nhanes.htm). And we selected covariates, including demographic data, comorbidities, lifestyle variables, height, BMI, WC, WHtR, TG, and HDL-C, based on statistical significance and therapeutic relevance.

### Statistical analysis

We used R statistical packages (The R Foundation; http://www.r-project.org; version 4.2.1) and EmpowerStats (www.empowerstats.net, X&Y solutions, Inc. Boston, Massachusetts) for all statistical analyses. We considered differences to be statistically significant at *P* < 0.05. Through the use of sample weights and a sophisticated sampling methodology, NHANES was able to acquire data that was nationally representative. We used the sample weight calculation method proposed by NHANES to weight the data in this paper.

We used mean ± SD to express continuous data, with the number data expressed as *n* (%). First, we compared the baseline characteristics of CMI quartile groups and CMI-age quartiles groups. The quintile cut-off values of the CMI are 0.27, 0.48, and 0.83. The quintile cut-off values of the CMI-age are 11.78, 23.13, and 43.88. Then, we used multiple logistic regression models to determine the relationship between CHF and CMI. We applied multivariate adjusted models and nonadjusted models. The variables were adjusted for age, sex, and race; education level, family PIR, and marital status; smoking status; moderate recreational activities; BMI, DM and hypertension status. We completed stratified and interaction analyses based on gender, age, and race; marital status; smoking status; moderate recreational activities; BMI, DM and hypertension status. Finally, we explored the associations of CMI and CMI-age with CHF using smooth curve plots. To assess the predictive efficacy of CMI and CMI-age for patients with CHF, we used receiver operating characteristic (ROC) curves.

## Results

### Baseline characteristics of study population

We extracted data of 9,008 participants from the NHANES database (Figure 1). Then, we divided the data into four groups by CMI quartile and CMI-age quartile. Table 1 lists the baseline characteristics for the analysis samples. CHF prevalence was 3.31%. We observed significant differences in demographic data among these CMI and CMI-age groups (e.g., race, gender, and age; marital status; education levels; and family PIR). In the higher CMI and CMI-age quartile groups, the proportion of smoking status increased and moderate recreational activities decreased. In addition, in the higher CMI and CMI-age quartile WC, BMI, WHtR, and TG tended to increase, whereas HDL-C decreased. Predictably, hypertension, diabetes, and CHF also increased in these groups. We also divided the data into two groups by CHF status, and the demographic data including gender, race, age, marital status, education levels, family PIR, smoking status, moderate recreational activities, BMI, WC, WHtR, TG, HDL-C, CMI, CMI-age hypertension status, and DM status showed significant differences between two groups (Supplementary Table S1).

**Table 1:** Characteristics of participants according to CMI quartile and CMI-age quartile.

Characteristics	CMI				*p-*value	CMI-age				*p-*value
	Q1 *N* = 2,252	Q2 *N* = 2,252	Q3 *N* = 2,252	Q4 *N* = 2,252		Q1 *N* = 2,252	Q2 *N* = 2,252	Q3 (0.48–0.83) *N* = 2,252	Q4 (0.83–4.01) *N* = 2,252	
Age (years)	45.83 ± 18.21	49.98 ± 17.64	51.79 ± 17.03	51.68 ± 15.87	<0.001	36.49 ± 14.64	48.90 ± 16.25	54.77 ± 15.63	59.11 ± 14.01	<0.001
Family PIR	2.65 ± 1.58	2.57 ± 1.57	2.42 ± 1.52	2.34 ± 1.50	<0.001	2.50 ± 1.66	2.64 ± 1.66	2.47 ± 1.62	2.37 ± 1.56	<0.001
BMI (kg/m^2^)	24.90 ± 5.11	28.45 ± 6.35	30.55 ± 6.69	32.78 ± 7.01	<0.001	25.24 ± 5.44	28.83 ± 6.83	30.28 ± 6.79	32.33 ± 6.72	<0.001
WHtR	0.53 ± 0.08	0.59 ± 0.09	0.62 ± 0.09	0.66 ± 0.10	<0.001	0.52 ± 0.08	0.59 ± 0.09	0.62 ± 0.09	0.66 ± 0.09	<0.001
Standing height (cm)	166.48 ± 9.46	167.09 ± 9.95	166.58 ± 10.21	167.73 ± 10.22	<0.001	167.46 ± 9.49	167.03 ± 9.94	166.60 ± 10.17	166.79 ± 10.28	0.025
Waist circumference (cm)	87.29 ± 12.46	97.70 ± 14.78	103.37 ± 14.75	109.63 ± 15.72	<0.001	87.10 ± 12.75	98.17 ± 15.23	103.21 ± 14.92	109.48 ± 14.91	<0.001
Direct HDL-cholesterol (mmol/L)	1.81 ± 0.44	1.47 ± 0.29	1.27 ± 0.24	1.06 ± 0.21		1.70 ± 0.46	1.49 ± 0.36	1.31 ± 0.29	1.10 ± 0.23	<0.001
Triglyceride (mmol/L)	0.61 ± 0.19	0.93 ± 0.23	1.30 ± 0.30	2.28 ± 0.84		0.65 ± 0.23	0.95 ± 0.30	1.31 ± 0.42	2.21 ± 0.88	<0.001
Sex, *n* (%)					<0.001					<0.001
Male	885 (39.30)	1,045 (46.40)	1,125 (49.96)	1,348 (59.86)		912 (40.55)	1,041 (46.16)	1,167 (51.82)	1,283 (56.97)	
Female	1,367 (60.70)	1,207 (53.60)	1,127 (50.04)	904 (40.14)		1,337 (59.45)	1,214 (53.84)	1,085 (48.18)	969 (43.03)	
Race/ethnicity, *n* ()					<0.001					<0.001
Mexican American	191 (8.48)	272 (12.08)	345 (15.32)	407 (18.07)		219 (9.74)	288 (12.77)	344 (15.28)	364 (16.16)	
Other Hispanic	167 (7.42)	224 (9.95)	295 (13.10)	286 (12.70)		197 (8.76)	209 (9.27)	267 (11.86)	299 (13.28)	
Non-Hispanic White	795 (35.30)	821 (36.46)	822 (36.50)	976 (43.34)		768 (34.15)	799 (35.43)	832 (36.94)	1,015 (45.07)	
Non-Hispanic Black	663 (29.44)	585 (25.98)	425 (18.87)	247 (10.97)		622 (27.66)	581 (25.76)	458 (20.34)	259 (11.50)	
Other Race, Including Multiracial	436 (19.36)	350 (15.54)	365 (16.21)	336 (14.92)		443 (19.70)	378 (16.76)	351 (15.59)	315 (13.99)	
Marital status					<0.001					<0.001
Married	1,037 (46.05)	1,119 (49.69)	1,214 (53.91)	1,266 (56.22)		889 (39.53)	1,161 (51.49)	1,284 (57.02)	1,302 (57.82)	
Widowed	142 (6.31)	162 (7.19)	163 (7.24)	154 (6.84)		50 (2.22)	146 (6.47)	190 (8.44)	235 (10.44)	
Divorced	203 (9.01)	264 (11.72)	246 (10.92)	265 (11.77)		154 (6.85)	244 (10.82)	271 (12.03)	309 (13.72)	
Separated	70 (3.11)	70 (3.11)	94 (4.17)	72 (3.20)		64 (2.85)	76 (3.37)	92 (4.09)	74 (3.29)	
Never married	603 (26.78)	432 (19.18)	346 (15.36)	309 (13.72)		827 (36.77)	398 (17.65)	260 (11.55)	205 (9.10)	
Living with partner	197 (8.75)	205 (9.10)	189 (8.39)	186 (8.26)		265 (11.78)	230 (10.20)	155 (6.88)	127 (5.64)	
Education level, *n* (%)					<0.001					<0.001
Less than 9th grade	128 (5.68)	200 (8.88)	215 (9.55)	270 (11.99)		90 (4.00)	180 (7.98)	238 (10.57)	305 (13.54)	
9th to 11th grade	232 (10.30)	272 (12.08)	339 (15.05)	341 (15.14)		227 (10.09)	275 (12.20)	351 (15.59)	331 (14.70)	
High school graduate or equivalent	463 (20.56)	498 (22.11)	511 (22.69)	515 (22.87)		461 (20.50)	505 (22.39)	507 (22.51)	514 (22.82)	
Some college or AA degree	698 (30.99)	678 (30.11)	661 (29.35)	685 (30.42)		759 (33.75)	662 (29.36)	630 (27.98)	671 (29.80)	
College graduate or above	731 (32.46)	604 (26.82)	526 (23.36)	441 (19.58)		712 (31.66)	633 (28.07)	526 (23.36)	431 (19.14)	
Smoked at least 100 cigarettes, *n* (%)					<0.001					<0.001
Yes	828 (36.77)	926 (41.12)	1,000 (44.40)	1,150 (51.07)		757 (33.66)	933 (41.37)	1,040 (46.18)	1,174 (52.13)	
No	1,424 (63.23)	1,326 (58.88)	1,252 (55.60)	1,102 (48.93)		1,492 (66.34)	1,322 (58.63)	1,212 (53.82)	1,078 (47.87)	
Diabetes status, *n* (%)					<0.001					<0.001
Yes	121 (5.37)	233 (10.35)	364 (16.16)	517 (22.96)		60 (2.67)	179 (7.94)	377 (16.74)	619 (27.49)	
No	2,131 (94.63)	2,019 (89.65)	1,888 (83.84)	1,735 (77.04)		2,189 (97.33)	2,076 (92.06)	1,875 (83.26)	1,633 (72.51)	
Hypertension status, *n* (%)										<0.001
Yes	572 (25.40)	787 (34.95)	915 (40.63)	1,058 (46.98)	<0.001	365 (16.23)	729 (32.33)	1,003 (44.54)	1,235 (54.84)	
No	1,680 (74.60)	1,465 (65.05)	1,337 (59.37)	1,194 (53.02)		1,884 (83.77)	1,526 (67.67)	1,249 (55.46)	1,017 (45.16)	
Moderate recreational activities					<0.001					<0.001
Yes	1,118 (49.64)	974 (43.25)	874 (38.81)	789 (35.04)		1,113 (49.49)	1,019 (45.19)	869 (38.59)	754 (33.48)	
No	1,134 (50.36)	1,278 (56.75)	1,378 (61.19)	1,463 (64.96)		1,136 (50.51)	1,236 (54.81)	1,383 (61.41)	1,498 (66.52)	
Congestive heart failure					<0.001					<0.001
Yes	41 (1.82)	60 (2.66)	77 (3.42)	120 (5.33)		21 (0.93)	54 (2.39	73 (3.24)	150 (6.66)	
No	2,211 (98.18)	2,192 (97.34)	2,175 (96.58)	2,132 (94.67)		2,228 (99.07)	2,201 (97.61)	2,179 (96.76)	2,102 (93.34)	

Mean ± SD for continuous variables: the *p*-value was calculated by the weighted linear regression model. (%) for categorical variables; the *p*-value was calculated by the weighted chi-square test. The quintile cut-off values of the CMI are 0.27, 0.48, and 0.83. The quintile cut-off values of the CMI-age are 11.78, 23.13, and 43.88.

Family PIR, the ratio of family income to poverty; BMI, body mass index; CMI, cardiometabolic index.

### Association between CMI, CMI-age and CHF

The relationships of CMI and CMI-age with CHF are shown in Table 2. When indicators were analyzed as continuous variables, CMI and CMI-age showed positive correlations with CHF in both the crude and adjusted models (all *P* < 0.05). When indicators were analyzed as categorical variables, it was found that in all models, the ORs of group Q4 was significantly different compared to Q1 (all *P* < 0.05), suggesting the risk of CHF is significantly increased with higher CMI and higher CMI-age. In both the crude and adjusted models, the overall trend showed that the higher CMI quartiles and higher CMI-age quartiles were strongly associated with the incidence of CHF.

**Table 2:** Association between CMI, CMI-age and congestive heart failure.

Exposure	OR (95% CI)		
	Model 1	Model 2	Model 3
	(*n* = 9,008)	(*n* = 9,008)	(*n* = 9,008)
CMI per unit increase	1.57 (1.34, 1.83) < 0.001	1.68 (1.41, 2.00) < 0.001	1.24 (1.01, 1.50) 0.035
CMI quartile			
Quartile 1	1.0	1.0	1.0
Quartile 2	1.48 (0.99, 2.21) 0.057	1.25 (0.83, 1.89) 0.278	1.21 (0.79, 1.86) 0.386
Quartile 3	1.91 (1.30, 2.80) < 0.001	1.58 (1.07, 2.35) 0.023	1.44 (0.94, 2.20) 0.095
Quartile 4	3.04 (2.12, 4.35) < 0.001	2.83 (1.94, 4.12) < 0.001	1.70 (1.11, 2.62) 0.016
*P* for trend	<0.001	<0.001	0.009
CMI-age per unit increase	1.01 (1.01, 1.02) < 0.01	1.01 (1.01, 1.01) < 0.001	1.01 (1.00, 1.01) < 0.001
CMI-age quartile			
Quartile 1	1.0	1.0	1.0
Quartile 2	2.60 (1.57, 4.32) < 0.001	1.29 (0.77, 2.17) 0.337	1.58 (0.92, 2.73) 0.098
Quartile 3	3.55 (2.18, 5.80) < 0.001	1.34 (0.80, 2.22) 0.267	1.38 (0.81, 2.37) 0.239
Quartile 4	7.57 (4.78, 12.00) < 0.001	2.48 (1.52, 4.06) < 0.001	1.97 (1.16, 3.34) 0.012
*P* for trend	1.86 (1.65, 2.09) < 0.001	1.39 (1.22, 1.59) < 0.001	1.20 (1.04, 1.38) 0.013

The quintile cut-off values of the CMI are 0.27, 0.48, and 0.83. The quintile cut-off values of the CMI-age are 11.78, 23.13, and 43.88.

Model 1: No covariates were adjusted.

Model 2: Age, sex, and race were adjusted.

Model 3: Age, sex, race/ethnicity, educational level, family PIR, diabetes status, marital status, BMI, hypertension status, smoking status, and moderate recreational activities were adjusted.

OR, odds ratio; 95% CI, 95% conﬁdence interval; family PIR, the ratio of family income to poverty; BMI, body mass index; CMI, cardiometabolic index.

### Subgroup analysis

We used sex, race, age, education level, marital status, smoking status, BMI, moderate recreational activities, hypertension, and diabetes as stratification variables, and performed stratified analysis to assess the effects of CMI and CMI-age on CHF. As shown in Table 3, associations of CMI and CMI-age with CHF were similar in all stratified populations (*P* for interaction > 0.05).

**Table 3:** Results of subgroup analysis and interaction analysis.

Subgroup	CMI			CMI-age		
	OR (95%CI)	*P*	*P* for interaction	OR (95%CI)	*P*	*P* for interaction
Sex			0.481			0.810
Male	1.21 (0.94, 1.55)	0.133		1.00 (1.00, 1.01)	0.214	
Female	1.39 (1.03, 1.87)	0.031		1.00 (1.00, 1.01)	0.199	
Age	** **	** **	0.398			0.916
20–59	1.08 (0.74, 1.58)	0.682		1.00 (1.00, 1.01)	0.263	
≥60	1.30 (1.04, 1.64)	0.023		1.00 (1.00, 1.01)	0.039	
Race/ethnicity			0.557			0.717
Mexican American	0.86 (0.41, 1.80)	0.686		1.00 (0.99, 1.01)	0.905	
Other Hispanic	1.00 (0.50, 2.00)	0.994		1.00 (0.99, 1.01)	0.868	
Non-Hispanic White	1.32 (1.02, 1.70)	0.032		1.00 (1.00, 1.01)	0.167	
Non-Hispanic Black	1.65 (1.06, 2.59)	0.028		1.01 (1.00, 1.01)	0.077	
Other Race, Including Multiracial	1.33 (0.65, 2.75)	0.435		1.00 (0.99, 1.02)	0.560	
Marital status			0.178			0.735
Married	1.32 (1.01, 1.73)	0.044		1.00 (1.00, 1.01)	0.017	
Widowed	1.43 (0.88, 2.31)	0.144		1.00 (1.00, 1.01)	0.099	
Divorced	1.15 (0.69, 1.92)	0.596		1.00 (1.00, 1.01)	0.234	
Separated	0.83 (0.13, 5.48)	0.849		1.00 (0.97, 1.02)	0.765	
Never married	1.74 (0.96, 3.18)	0.070		1.01 (1.00, 1.02)	0.015	
Living with partner	0.07 (0.00, 1.50)	0.088		1.00 (0.98, 1.02)	0.904	
Education level			0.915			0.916
Less than 9th grade	1.09 (0.62, 1.91)	0.756		1.00 (1.00, 1.01)	0.444	
9th 11th grade	1.17 (0.71, 1.93)	0.531		1.00 (1.00, 1.01)	0.203	
High school graduate or equivalent	1.43 (0.99, 2.06)	0.058		1.00 (1.00, 1.01)	0.034	
Some college or AA degree	1.38 (0.97, 1.97)	0.075		1.00 (1.00, 1.01)	0.009	
College graduate or above	1.20 (0.65, 2.22)	0.556		1.00 (0.99, 1.01)	0.502	
Smoking status	** **	** **	0.665			0.863
Yes	1.18 (0.92, 1.52)	0.053		1.01 (1.00, 1.01)	0.005	
No	1.17 (0.85, 1.61)	0.344		1.00 (1.00, 1.01)	0.042	
Diabetes status	** **	** **	0.874			0.342
Yes	1.21 (0.91, 1.62)	0.189		1.00 (1.00, 1.01)	0.081	
No	1.25 (0.96, 1.63)	0.098		1.01 (1.00, 1.01)	0.002	
BMI (kg/m2)	** **	** **	0.828			0.853
<25	1.25 (0.63, 2.46)	0.522		1.01 (1.00, 1.02)	0.185	
≥25, <30	1.35 (0.94, 1.94)	0.100		1.01 (1.00, 1.01)	0.048	
≥30	1.18 (0.92, 1.52)	0.195		1.00 (1.00, 1.01)	0.043	
Hypertension status			0.619			0.739
Yes	1.26 (1.01, 1.57)	0.039		1.00 (1.00, 1.01)	0.003	
No	1.12 (0.71, 1.76)	0.636		1.01 (1.00, 1.01)	0.075	
Moderate recreational activities	** **	** **	0.645			0.586
Yes	1.14 (0.77, 1.70)	0.510		1.00 (1.00, 1.01)	0.219	
No	1.26 (1.01, 1.58)	0.041		1.01 (1.00, 1.01)	0.001	

Age, gender, race/ethnicity, educational level, family PIR, diabetes status, marital status, hypertension status, smoking status, and moderate recreational activities were adjusted.

In the subgroup analysis, the model is not adjusted for the stratification variable.

OR, odds ratio; 95% CI, 95% conﬁdence interval; Family PIR, the ratio of family income to poverty; BMI, body mass index; CMI, cardiometabolic index.

### Nonlinear associations and ROC curves

**Figure 2:**
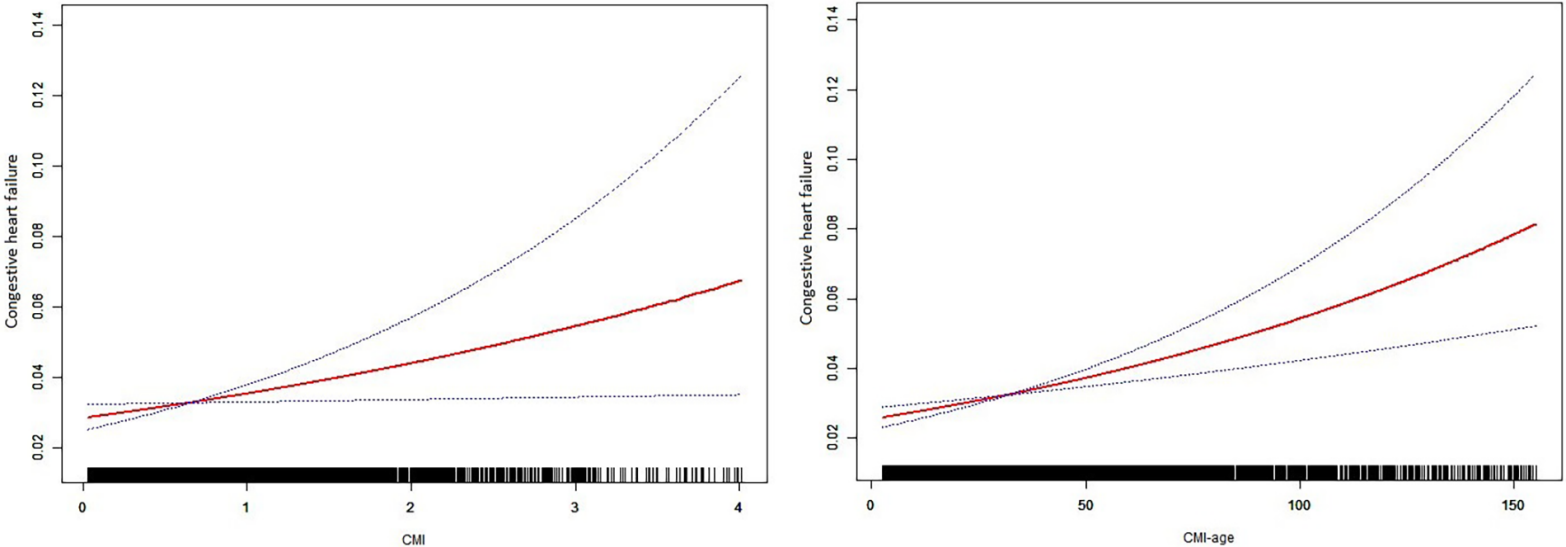
Smooth curve plots between CMI and heart failure. CMI, cardiometabolic index. Age, sex, race/ethnicity, educational level, family PIR, diabetes status, marital status, BMI, hypertension status, smoking status, and moderate recreational activities were adjusted.

**Figure 3:**
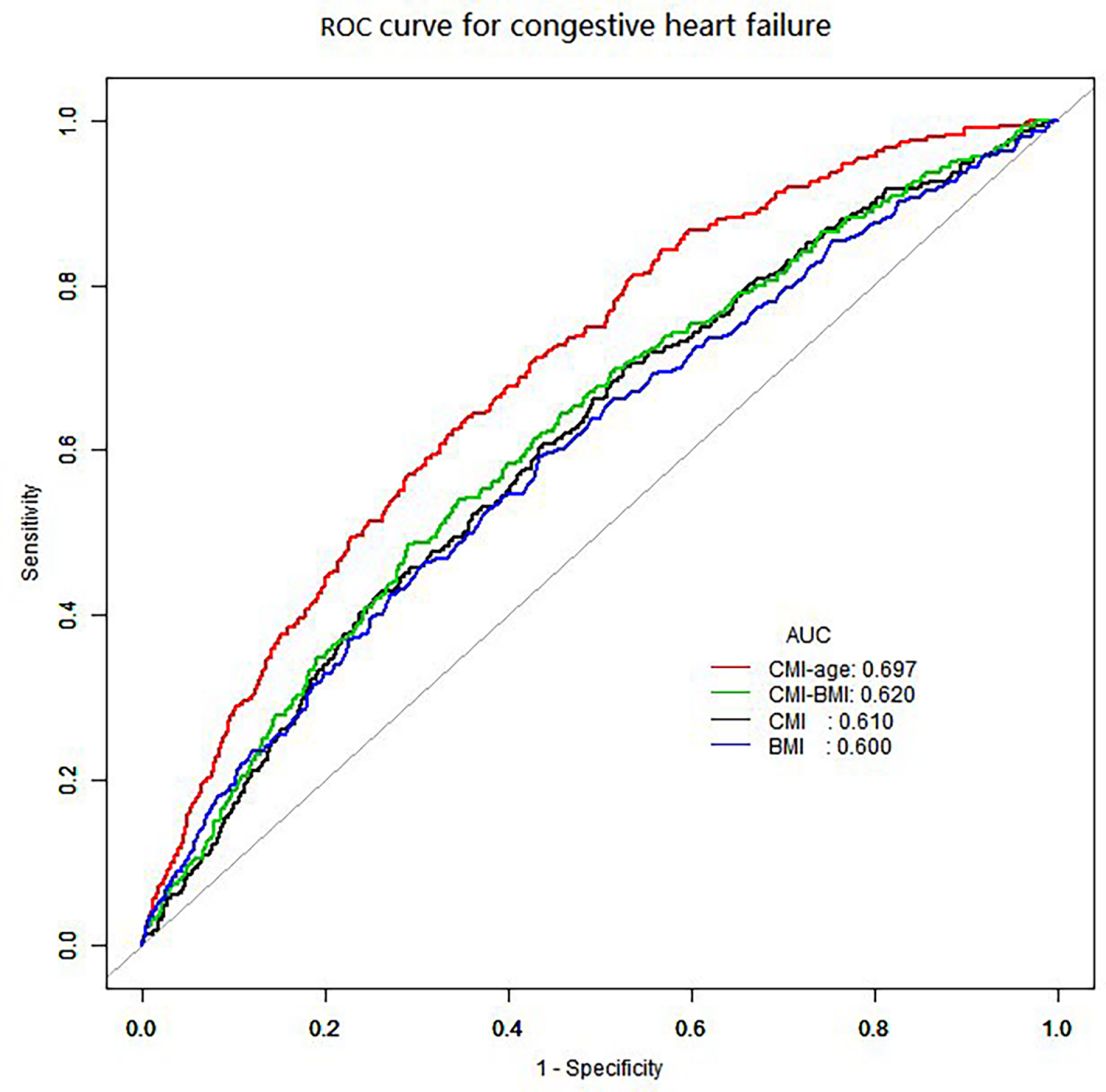
ROC curves for the identification of heart failure. CMI: AUC = 0.610 (95% CI, 0.578–0.642, specificity 0.472, sensitivity 0.701). CMI-age: AUC = 0.697 (95% CI, 0.668–0.725, specificity 0.648, sensitivity 0.638). BMI: AUC = 0.600 (95% CI, 0.566–0.633, specificity 0.565, sensitivity 0.593). CMI-BMI: AUC = 0.620 (95% CI, 0.588–0.653, specificity 0.710, sensitivity 0.485). CMI, cardiometabolic index; ROC, receiver operating characteristic curve; AUC, ROC area.

We also used smooth curve plots to examine the associations of CMI and CMI-age with CHF (Figure 2). To distinguish CHF, we analyzed the ROC curves for the CMI and CMI-age (Figure 3). Among the entire cohort, the area under the ROC curve (AUC) of the CMI for the identification of CHF was 0.610 (95% CI: 0.578–0.642, *P *< 0.001), the AUC of the CMI-age for the identification of CHF was 0.697 (95% CI, 0.668–0.725, *P *< 0.001). The AUC of the CMI-age was significantly higher than CMI (*P *< 0.001). Furthermore, we compared the AUCs of the CMI, CMI-age and BMI for the identification of CHF (Figure 3), suggesting that CMI-age was significantly better than CMI, BMI and CMI-BMI in predicting CHF (*P *< 0.001).

## Discussion

We evaluated the associations of CMI and CMI-age with the risk of CHF in US adults according to NHANES data for 2011–2018. The results showed that the overall prevalence of CHF was 3.31%, which was consistent with previous studies (6, 14). We provided evidence that CMI and CMI-age were independently associated with CHF and exhibited near-linear dose-response relationships. A deeper understanding of CMI, CMI-age and CHF in various populations also can be gained through subgroup analysis, which indicated that the direction of correlations between CMI, CMI-age and CHF in various subgroups was consistent with those in the study population as a whole.

Conventional risk factors (e.g., age, smoking, obesity, hypertension, and DM) are associated with CHF, according to population-based research (4, 17). Although obesity has posed an independent risk factor for CHF, the diversity of obesity phenotypes may result in differences in incidence, treatment outcomes, and mortality of CHF (18, 19). Recent literature has shown that increases in visceral adipose tissue (VAT) rather than subcutaneous fat are a significant risk factor for the development of CHF (20–22). This may due to the higher degrees of adipocyte hypertrophy, free fatty acids elevation, and insulin resistance among patients with increased VAT (23). CMI is now being used to evaluate VAT because it is noninvasive index that combines TG/HDL-C and WHtR (9). To measure abdominal obesity, WHtR is a valuable parameter and helps to identify cardiovascular disease (24, 25). To identify metabolic disorders and CVDs, TG/HDL-C is suitable (26–28). As a result, we proposed the use of CMI to identify CHF in adults.

Previous studies have concluded that CMI is associated with DM, CVDs, and MetS (9–15). Data are limited, however, on the relationship between the risk of CHF and CMI. We believe we are the first to examine the correlation between CMI and CHF in a significant sample of US adults. The logistic regression analysis showed that higher CMI quartiles were associated independently with elevated risk factors for CHF. We observed positive and robust correlations between CHF and CMI, regardless of multiple confounding factors. The ROC analysis showed that the diagnostic capacity of CMI was adequate, with an AUC of 0.610 (95% CI: 0.578–0.642). Together, these findings highlighted the clinical value of CMI for screening CHF among US adults. Previous studies also have found a strong correlation between CHF and VAT, which is consistent with our results (6, 20, 21). Past studies have measured VAT with magnetic resonance imaging and abdominal computed tomography. These methods, although accurate, are expensive and inefficient. Ye et al. demonstrated that CMI was independently correlated with left ventricular diastolic dysfunction among asymptomatic Chinese adults (10). Similar to our findings, it was also found that the diagnostic capacity of CMI was moderate and had an AUC of 0.615 (95% CI: 0.587–0.643) (10). Along a complex path from risk to fully developed CHF, a booming number of proteins related to damage, remodeling, and neurohormonal activation have been discovered (2). However, CMI can be calculated by measuring TG, HDL-C, height, and WC, which are simple and easy to obtain.

According to data from the European Society of Cardiology, 10% CHF patients are over 70 years old (16). The number of CHF is rising as a result of aging, an increase in the load of comorbidities and risk factors for the condition, and longer survival times following myocardial infarction (1–3, 16). Besides, a Japanese population based study showed that CMI and its association with DM are potently influenced by age (29). Thus, we proposed that a combination of CMI with age may potentially identify CHF more strongly. We evaluated the association between CMI-age and CHF, and compared indicators of CMI and CMI-age for early identification of CHF. The ROC curve investigation affirmed that CMI-age is a favorable surrogate indicator of CHF, and CMI-age could be an effective way to detect CHF during primary care examinations (30, 31).

## Strengths and limitations

The present study demonstrated that the CMI-age can be utilized as predictive tool for assessing the likelihood of developing CHF. And this finding establishes a foundation for promoting health and implementing preventive measures to manage and control CHF. However, the present study still has some limitations. Firstly, this was an observational study. This established the associations of CMI and CMI-age with CHF without establishing a causative relationship. Secondly, it is challenging to determine the severity of CHF due to limitations of the NHANES database. Thirdly, this study was conducted among adult Americans and had a limited population size, thereby limiting the generalizability of the results to CHF population from different geographical areas.

## Conclusion

The present study found that CMI and CMI-age were all independently correlated with CHF risk. Furthermore, high CMI and CMI-age warranted greater attention to prevent CHF risk. By combining biochemical and anthropometric lipid parameters, this novel index could be an effective way to detect CHF. The CMI-age index was significantly better than CMI in predicting CHF, which provides new insights into the prevention and management of CHF. CMI-age could be an effective way to detect CHF during primary care examinations, most notably in areas with limited medical resources.

## Data Availability

The original contributions presented in the study are included in the article/Supplementary Material, further inquiries can be directed to the corresponding author.
