# Supplementary material for: Association between cardiometabolic index and congestive heart failure among US adults: a cross-sectional study
Source: Front Cardiovasc Med. 2024 Sep 10;11:1433950. doi: 10.3389/fcvm.2024.1433950 (PMC11419996; doi:10.3389/fcvm.2024.1433950)
Supplement: Supplementary file 1 [file Table1.pdf]

Supplemenary Table 1. Basic characteristics between the two groups with and without congestive heart failure

**Characteristics**	**Non-congestive heart failure** **N=** 8704	**Congestive heart failure** N= 295	***p*-value**
Age(years)	49.26 ± 17.24	66.28 ± 12.53	<0.001
Family PIR	2.51 ± 1.63	2.00 ± 1.41	<0.001
BMI (kg/m^2)	29.08 ± 6.90	31.85 ± 8.33	<0.001
WHtR	0.59 ± 0.10	0.66 ± 0.11	<0.001
Standing Height (cm)	167.00 ± 9.98	166.23 ± 9.99	0.193
Waist Circumference (cm)	99.17 ± 16.51	109.09 ± 17.64	<0.001
Direct HDL-Cholesterol (mmol/L)	1.40 ± 0.41	1.28 ± 0.39	<0.001
Triglyceride (mmol/L)	1.27 ± 0.78	1.39 ± 0.76	0.017
CMI	0.65 ± 0.57	0.84 ± 0.65	<0.001
CMI-age	32.59 ± 31.44	55.84 ± 44.75	<0.001
**Sex, n (%)**			0.091
Male	4243 (48.71)	160 (53.69)	
Female	4467 (51.29)	138 (46.31)	
**Race/ethnicity, n (%)**			<0.001
Mexican American	1187 (13.63)	28 (9.40)	
Other Hispanic	939 (10.78)	33 (11.07)	
Non-Hispanic White	3266 (37.50)	148 (49.66)	
Non-Hispanic Black	1849 (21.23)	71 (23.83)	
Other Race - Including Multi-Racial	1469 (16.87)	18 (6.04)	
**Marital status**			<0.001
Married	4496 (51.62)	140 (46.98)	
Widowed	564 (6.48)	57 (19.13)	
Divorced	925 (10.62)	53 (17.79)	
Separated	295 (3.39)	11 (3.69)	
Never married	1663 (19.09)	27 (9.06)	
Living with partner	767 (8.81)	10 (3.36)	
**Education level, n (%)**			<0.001
Less than 9th grade	4496 (51.62)	140 (46.98)	
9-11th grade	564 (6.48)	57 (19.13)	
High school graduate or equivalent	925 (10.62)	53 (17.79)	
Some college or AA degree	295 (3.39)	11 (3.69)	
College graduate or above	1663 (19.09)	27 (9.06)	
**Smoked at least 100 cigarettes, n (%)**			<0.001
Yes	3724 (42.76)	180 (60.40)	
No	4986 (57.24)	118 (39.60)	
**Hypertension status, n (%)**			<0.001
Yes	3093 (35.51)	239 (80.20)	
No	5617 (64.49)	59 (19.80)	
**Diabetes status, n (%)**			
Yes	1114 (12.79)	121 (40.60)	<0.001
No	7596 (87.21)	177 (59.40)	
**Moderate recreational activities, n (%)**			<0.001
Yes	3677 (42.22)	78 (26.17)	

No	5033 (57.78)	220 (73.83)

Mean ± SD for continuous variables and n (%) for categorical variables.

Abbreviation: family PIR, the ratio of family income to poverty; BMI, body mass index; CMI, cardiometabolic index.
